# Nitroxoline is a novel inhibitor of NLRP3-dependent pyroptosis

**DOI:** 10.1038/s41420-025-02699-z

**Published:** 2025-08-20

**Authors:** Christina Maeder, Roberto Baumann, Susanne Gaul, Sven Fikenzer, Michael Schaefer, Hermann Kalwa, Ulrich Laufs, Alexander Kogel

**Affiliations:** 1 Klinik und Poliklinik für Kardiologie, Universitätsklinikum Leipzig, Leipzig, Germany; 2 Rudolf-Boehm-Institut für Pharmakologie und Toxikologie, Universität Leipzig, Leipzig, Germany

**Keywords:** Drug screening, Drug development

## Abstract

Aberrant activity of the NLR family pyrin domain containing 3 (NLRP3) inflammasome contributes to a wide range of diseases associated with acute inflammatory responses and chronic sterile inflammation. Activation of the NLRP3 inflammasome mediates pyroptotic cell death and the release of pro-inflammatory cytokines. To date, no selective inhibitor of inflammasome activity is available for the use in humans. We conducted a medium-throughput screening of 6280 drugs or drug-like compounds and identified novel inhibitors of the NLRP3 inflammasome. Among these, nitroxoline was further characterized because the drug is approved for antibiotic treatment in humans, and we found no toxicity over a wide range of concentrations. Treatment of THP-1 monocytes with 80 μM nitroxoline markedly reduced the secretion of the pro-inflammatory cytokine Interleukin-1β (IL-1β) by 95% from 197.8 pg ml^−1^ to 11.0 pg ml^−1^. Nitroxoline reduced downstream events of inflammasome activation including caspase-1 activity (FAM-Flica^+^/7AAD^+^ cells control 57.1 ± 9.4% vs. nitroxoline 27.9 ± 15.5%) and gasdermin D cleavage (ratio cleaved/uncleaved control 8.7 ± 4.3 vs. nitroxoline 1.3 ± 1.3, *p* < 0.05). The data were confirmed in cultured human PBMC, where nitroxoline abrogated IL-1β secretion. Mechanistically, drug affinity-responsive target assays revealed that nitroxoline directly interacts with the NACHT domain of NLRP3, inhibiting inflammasome assembly. Nitroxoline did not affect NF-κB-dependent gene expression, as analyzed by nuclear p65 translocation and IκBα phosphorylation, and did not inhibit the NLR-family member NLRC4 or the AIM2 inflammasomes, indicating specificity for NLRP3. Nitroxoline is a novel inhibitor of the NLRP3 inflammasome, which reduces inflammasome assembly and IL-1β release. These data set the stage for testing the effects of nitroxoline on sterile inflammation in clinical studies.

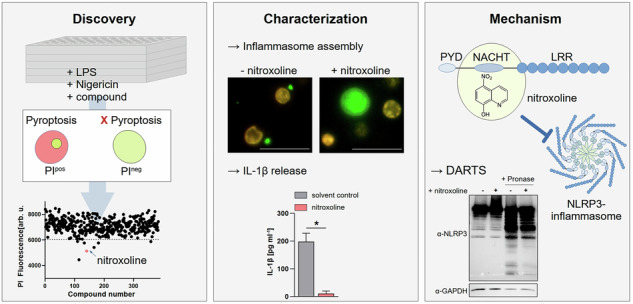

## Introduction

Inflammasomes are cytosolic sensors of pathogen-derived activating signals (PAMPs) and danger-derived activating signals (DAMPs) that contribute to the first line of the immune response. The assembly of inflammasomes leads to the activation of caspase-1, which mediates the cleavage of IL-1β, IL-18, and gasdermin D [1–4].

The NLRP3 inflammasome is a multiprotein complex consisting of NLRP3 and caspase-1 linked by the apoptosis-associated speck-like protein containing card (ASC). The N-terminal PYRIN (PYD), central NAIP, CIITA, HET-E, and TP1 (NACHT), and C-terminal leucine-rich repeat (LRR) domains comprise the three functional domains of the NLRP3 protein [5]. The PYD domain facilitates interaction with the ASC protein, functioning as an adapter between NLRP3 and caspase-1 [6]. NACHT contains an ATPase that mediates conformational changes from an autoinhibited to an active state [7, 8]. The LRR domain exerts a regulatory function, acting as a site for post-translational modifications and promoting the formation of the characteristic double-ring cage structure of the active NLRP3 inflammasome via self-interaction [9–12].

The activation of the NLRP3 inflammasome includes a “priming” and an “activation” step. Transcriptional priming by toll-like receptor stimulation or cytokines induces the NF-κB-dependent expression of *IL1B*, *IL18*, and *NLRP3* and post-translational modifications [13–15]. The activation signal comprises changes in potassium efflux triggered by different bacterial species and other ion-derived stimuli, such as mitochondrial reactive oxygen species [16]. Solid particles, such as cholesterol crystals, l-leucyl-l-leucine methyl ester (a product of lysosomal damage) [17], and misfolded proteins, as observed in Alzheimer’s disease [18] are also potent activators.

NLRP3 dysfunction is present in multiple diseases. A gain-of-function mutation in the *NLRP3* gene leads to cryopyrin-associated periodic fever syndrome and Muckle-Wells syndrome. NLRP3 is an important mediator of sterile inflammatory diseases, including gout, type 2 diabetes, and vascular diseases such as atherosclerosis. However, a specific treatment for inflammasome-related disorders is still not available [19–21].

Over the last decade, several NLRP3 inhibitors were identified. One promising candidate was MCC950, a potent blocker of the ATPase activity of NLRP3 [22, 23]. Clinical trials with MCC950 have been discontinued because of hepatotoxicity. Another candidate is CY-09, which interacts directly with NLRP3 and inhibits ATP binding [24]. Recently, alantolactone, a compound used in Chinese medicine, has been shown to inhibit inflammasome assembly by binding to NLRP3’s NACHT domain [25]. However, to date no compound has received regulatory approval for the use in humans.

In this study, we characterized nitroxoline, a candidate identified in medium-throughput screening and already approved as an antibiotic for short- and long-term use in humans, as a novel NLRP3 inhibitor.

## Results

**Fig. 1:**
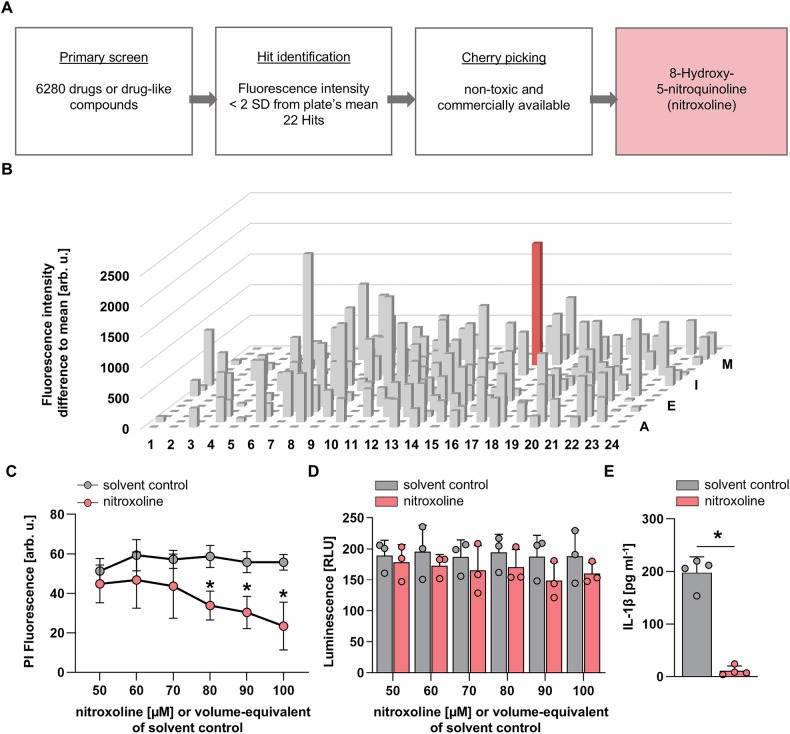
Compound library screening identifies nitroxoline as a novel inhibitor of pyroptosis. **A** Workflow for target identification screening of the Selleckchem Bioactive Compound Library and Spectrum library, comprising 6280 drugs, drug-like compounds, and natural compounds. **B** Exemplary results of the screening of a 384-well plate. Propidium iodide (PI) fluorescence of THP-1 ASC-GFP cells after drug treatment and incubation with 1 µg ml^−1^ LPS (4 h) and 5 µg ml^−1^ nigericin (1 h), assayed at 20 µM for each compound. Propidium iodide fluorescence intensity difference to mean is presented. Nitroxoline is shown in red. **C** Concentration-response curve of LPS- and nigericin-treated THP-1 cells depicting propidium iodide (PI) fluorescence at the indicated compound concentrations. DMSO volume was adjusted to that of nitroxoline (*n* = 6). **D** Cell viability was analyzed using the CellTiter-Glo assay of nitroxoline-treated THP-1 WT cells at the indicated concentrations. The volume of DMSO was adjusted to nitroxoline concentrations (*n* = 3). **E**: IL-1β release from THP-1 WT cells treated with LPS and nigericin with the addition of 80 µM nitroxoline or equal volume of DMSO as solvent control (*n* = 4). Data are presented as mean + SD. Data were analyzed using the Student’s *t*-test. *p < 0.05.

Nitroxoline was identified as novel inhibitor of pyroptosis by medium-throughput screening of the Selleckchem Bioactive Compound library and the Spektrum Library (Fig. 1A). The screening of 6280 drugs and drug-like compounds identified nitroxoline as a novel inhibitor of pyroptotic cell death in THP-1 ASC-GFP cells (Fig. 1B). Wild-type THP1 cells were used for validation experiments, unless otherwise stated. At concentrations of 80–100 µM, nitroxoline significantly reduced propidium iodide (PI) fluorescence in THP-1 cells compared to DMSO-treated cells (Fig. 1C). Compound toxicity was analyzed using the CellTiter-Glo assay, which indicates metabolically active cells. None of the tested nitroxoline concentrations were toxic when applied for 4 h in culture medium (Fig. 1D). Treatment of THP-1 monocytes with 80 µM nitroxoline markedly reduced IL-1β secretion from 197.8 pg ml^−1^ to 11.0 pg ml^−1^ (*p* < 0.001, Fig. 1E).

### Nitroxoline inhibits NLRP3 inflammasome assembly

**Fig. 2:**
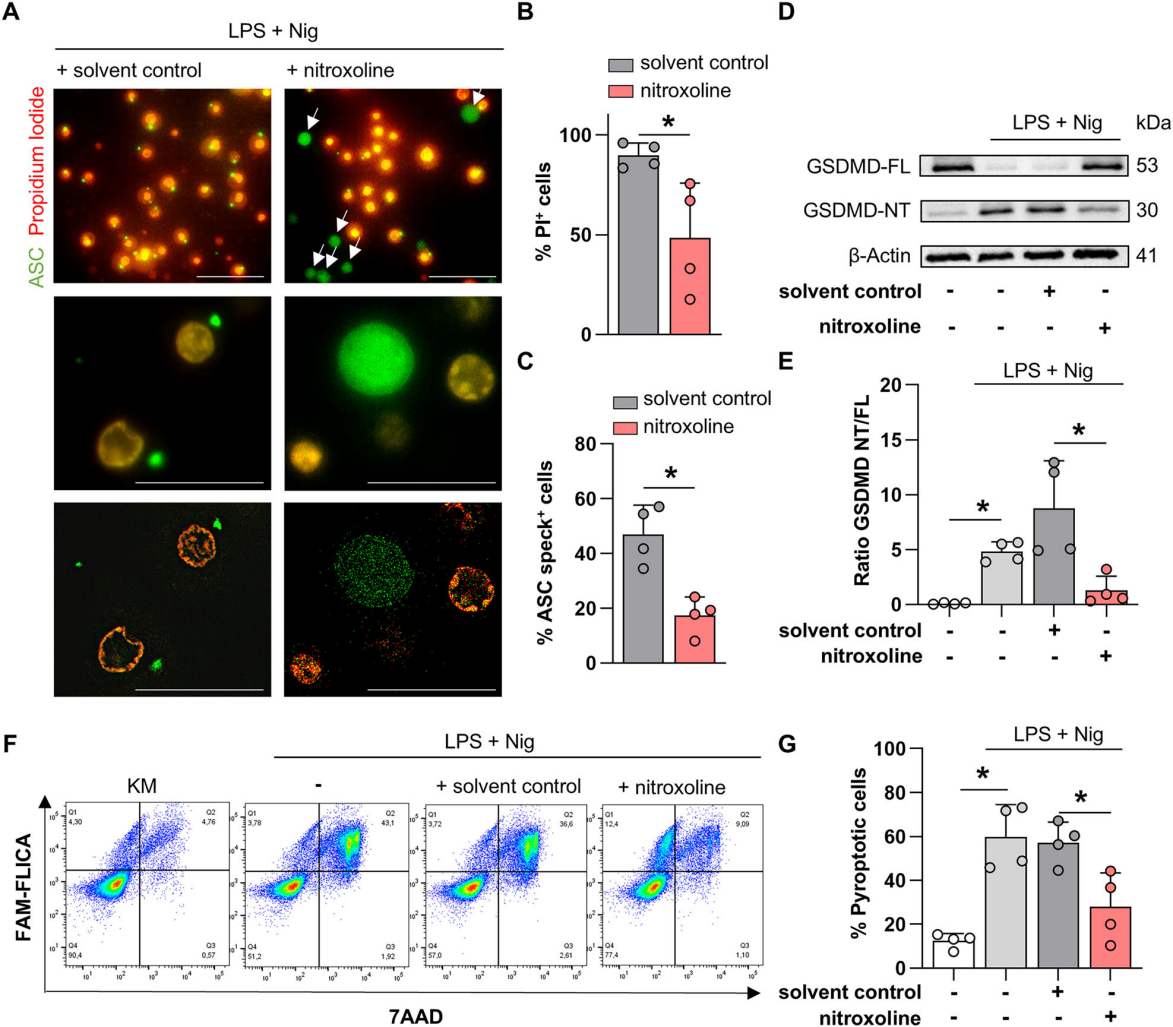
Nitroxoline inhibits inflammasome assembly. LPS-primed THP-1 ASC-GFP cells were treated with the indicated compound for 3 h before the addition of nigericin (Nig) for 1 h to examine ASC-speck formation. Next, 80 µM nitroxoline or an equivalent volume of DMSO was added. **A** Representative images of unfixed THP-1 ASC-GFP cells treated as indicated. ASC-GFP is depicted in green, and propidium iodide-positive cells are shown in red. Arrowheads indicate primed, non-pyroptotic cells. Cells were imaged at 32 × magnification, scale bar: 100 µm (top row). Unprocessed (central row) and SR-SIM-processed (bottom row) images of THP-1 ASC-GFP cells treated as indicated at 100 × magnification, scale bar 20 µm. Bar plots depicting the percentage of propidium iodide-positive (PI + ) (**B**) and ASC speck-positive (ASC speck + ) cells (**C**) assessed by flow cytometry (n = 4). **D, E** Representative Immunoblot of THP-1 WT cells treated as indicated. Full-length (GSDM-FL) and cleaved (GSDMD-NT) gasdermin D are presented with β-actin as a loading control (**D**). Bar plots presenting the ratio of GSDMD-NT to GSDM-FL (*n* = 4) (**E**). **F, G** Representative flow cytometry scatter plots of THP-1 cells treated as indicated. Quarter 2 (Q2) represents the FAM-FLICA, 7AAD-double positive pyroptotic cell population (**F**). Corresponding bar plot depicting the percentage of pyroptotic cells measured in Q2 (*n* = 4) (**G**). Data are presented as mean + SD. Data were analyzed using Student’s *t*-test. *p < 0.05.

Treatment with nitroxoline along with LPS and nigericin reduced ASC speck formation in THP-1 ASC-GFP cells, leaving the cells in a “primed” state (Fig. 2A). Flow cytometry analysis revealed a significant reduction in PI-positive cells by 44.9% (*p* < 0.05, Fig. 2B) and ASC speck-positive cells by 63.1% (*p* < 0.05, Fig. 2C). Gasdermin-D cleavage significantly increased after LPS and nigericin treatment, as quantified by the ratio of N-terminal protein fragment (GSDMD-NT) to full-length gasdermin D (GSDMD-FL). Nitroxoline abrogated the effect of LPS and nigericin treatment on the gasdermin D NT/FL ratio, whereas the addition of DMSO had no such impact (Fig. 2D, E). Pyroptotic cells were identified as a 7AAD-FAM-FLICA double-positive population by flow cytometry. LPS and nigericin treatment significantly increased the fraction of pyroptotic cells from 12.5% to 59.9% (*p* < 0.05). Nitroxoline significantly reduced the number of pyroptotic cells to 27.9% compared to DMSO control (*p* < 0.05, Fig. 2H–I).

### Nitroxoline reduces IL-1β release from human peripheral blood mononuclear cells ex vivo

**Fig. 3:**
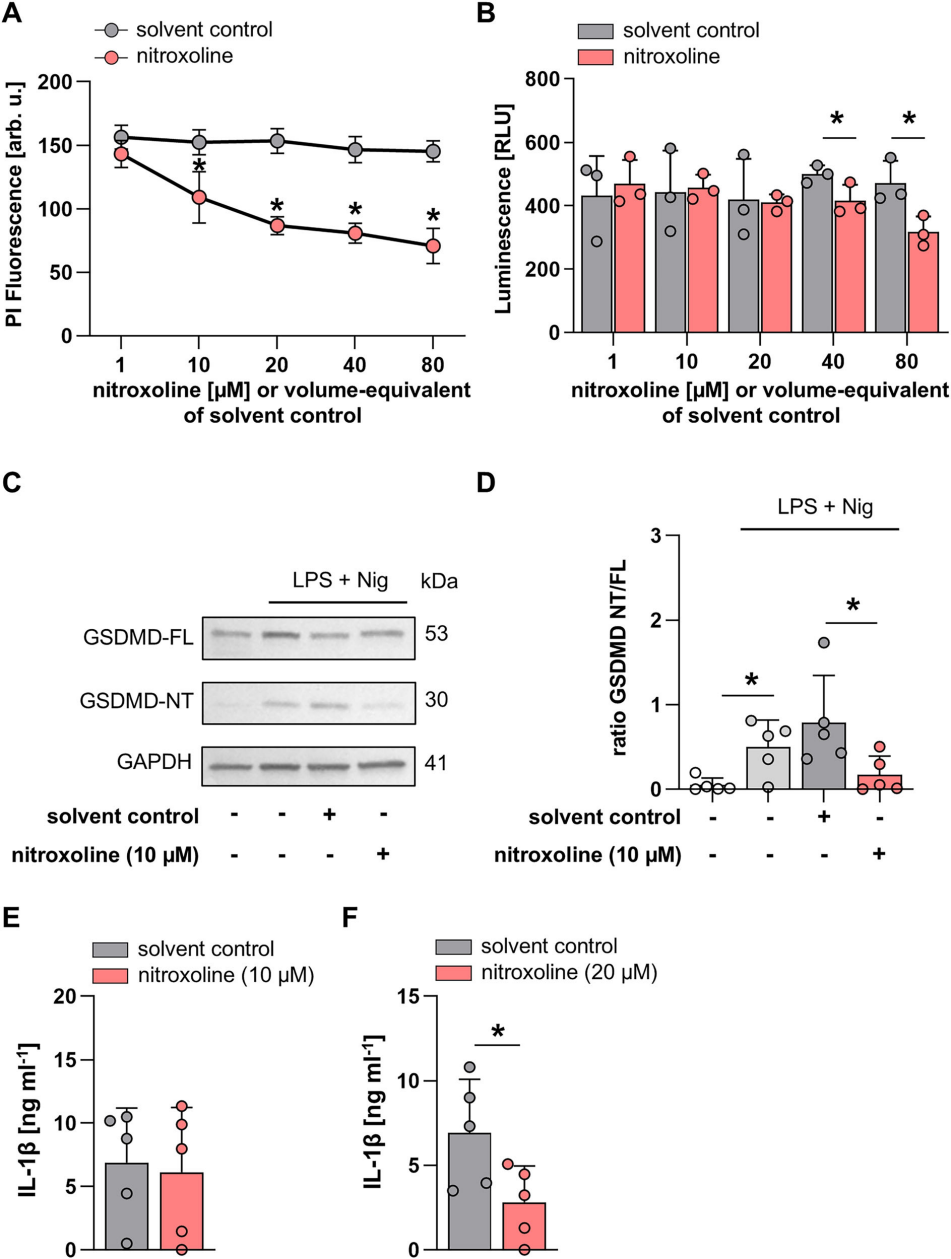
Nitroxoline alleviates IL-1β release from PBMC ex vivo. **A** Concentration-response curve of LPS- and nigericin-treated PBMC depicting propidium iodide (PI) fluorescence at the indicated compound concentrations. The volume of DMSO was adjusted to that of nitroxoline (*n* = 6). **B** Cell viability was analyzed using the CellTiter-Glo assay of nitroxoline-treated PBMC at the indicated concentrations. DMSO volume was adjusted to nitroxoline concentrations (*n* = 3). **C, D** Representative immunoblot of PBMC treated as indicated with 10 µM nitroxoline or equivalent volume of DMSO. Full-length (GSDMD-FL) and cleaved (GSDMD-NT) gasdermin D are presented with GAPDH as a loading control (**C**). Bar plots presenting the ratio of GSDMD-NT to GSDMD-FL (n = 5, **D**). Secreted IL-1β (ng ml^−1^) in the supernatants of LPS- and nigericin-treated PBMC with the addition of either 10 µM (**E**) or 20 µM (**F**) nitroxoline and the equivalent volume of DMSO (*n* = 5). Data are presented as mean + SD. Data were analyzed using Student’s *t*-test. *p < 0.05.

In cultured PBMC, 10 µM nitroxoline was sufficient to inhibit pyroptotic cell death (Fig. 3A). Concentrations ranging from 10 to 20 µM did not affect cell viability, as measured by quantification of intracellular ATP levels (Fig. 3B). 10 µM nitroxoline significantly reduced gasdermin D cleavage in LPS- and nigericin-treated PBMC compared to LPS- and nigericin-treated PBMC with the addition of DMSO (−65.5%, *p* < 0.05) (Fig. 3C, D). Although 10 µM nitroxoline was sufficient to reduce gasdermin D cleavage, this effect was not reflected in IL-1β secretion from LPS- and nigericin-treated PBMC (Fig. 3E). An increase to 20 µM nitroxoline resulted in a significant reduction in IL-1β concentration in the supernatant of LPS- and nigericin-treated PBMC (6.9 vs. 2.8 ng ml^−1^, *p* < 0.05) (Fig. 3F). Of note, the effective concentration of 20 µM to inhibit IL-β release from isolated human PBMC is comparable to the serum concentrations of nitroxoline achieved during the treatment of urinary tract infections [26].

### Nitroxoline does not affect the NF-κB signaling pathway

**Fig. 4:**
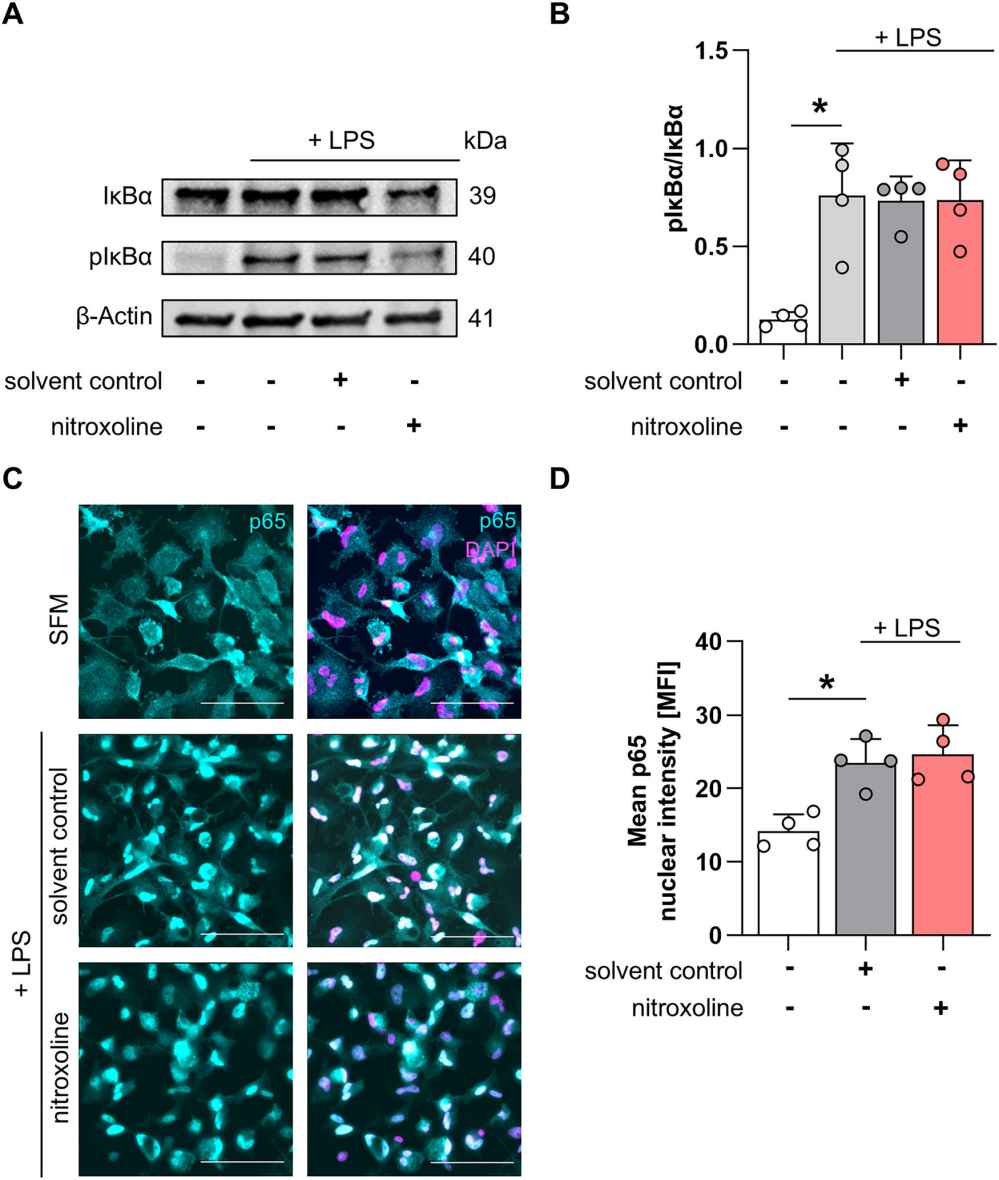
Nitroxoline does not inhibit the NF-κB pathway. **A** Immunblot presenting IkBα and phosphorylated IkBα (pIkBα) levels in LPS-primed THP-1 cells treated with 80 µM nitroxoline or solvent control. β-Actin served as loading control. **B** Corresponding bar plots presenting the pIκBα/IκBα ratio (*n* = 4). **C** Representative images of p65 nuclear translocation in PMA-differentiated THP-1 macrophages. LPS-primed Macrophages were treated with 80 µM nitroxoline or DMSO. Cells were imaged at 32 × magnification, with a scale bar of 100 µm. p65 is presented in cyan and nuclei in magenta. **D** Barplot depicting the nuclear mean p65 fluorescence intensity; acquisition parameters were unchanged between measurements (*n* = 4). Data are presented as mean + SD. Data were analyzed using Student’s *t*-test. *p < 0.05.

To determine whether nitroxoline interferes with the priming or activation step of the inflammasome cascade, the phosphorylation of IκBα and translocation of p65 to the nucleus following NF-κB activation were measured. Treatment of THP-1 cells with LPS significantly increased the ratio of phosphorylated IkBa (pIκBα) to IκBα from 0.1 to 0.8, as determined by western blotting. The addition of DMSO or 80 µM nitroxoline did not reduce pIκBα levels (Fig. 4A, B, Supplementary Fig. 1A). Treatment of THP-1 macrophages with LPS increased p65 translocation from the cytoplasm to the nucleus from 14.2 to 23.5 relative fluorescence units (RFU), which was not changed by nitroxoline (24.7 RFU, Fig. 4C, D).

### Nitroxoline inhibits inflammasome assembly by binding NLRP3 inflammasome

**Fig. 5:**
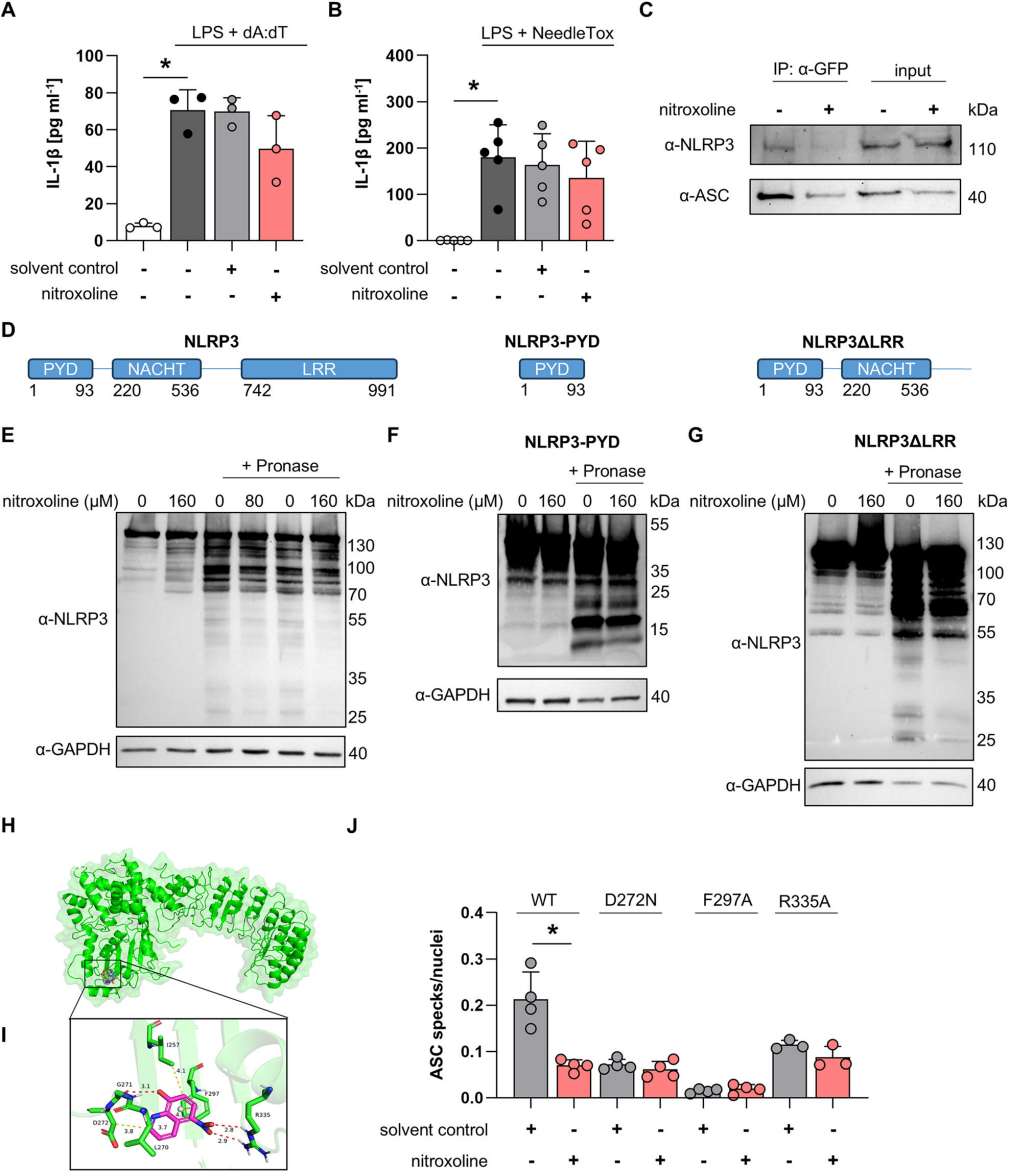
Nitroxoline inhibits NLRP3 by targeting the NACHT domain. IL-1β release from THP-1 WT cells treated with 1 µg ml^−1^ LPS and 3 µg ml^−1^ polydA:dT (n = 3, **A**) or 1 µg ml^−1^ LPS and NeedleTox (n = 5, **B**). Nitroxoline and the solvent control were added as indicated. **C** Co-immunoprecipitation from THP-1 ASC-GFP cells treated with 1 µg ml^−1^ LPS for 3 h and 5 µg ml^−1^ nigericin using α-GFP antibody. 120 µM nitroxoline and solvent control were added as indicated. **D** Schematic representation of the domains present in full-length NLRP3 and the deletion constructs NLRP3-PYD and NLRP3ΔLRR. **E** DARTs assay of pEGFP-C2-NLRP3-transfected HEK293 cell lysate incubated with the indicated concentration of nitroxoline or solvent control digested with pronase (28 ng µg^−1^) for 30 min and analysed by immunoblot. DARTs assay of HEK293 cell lysates transfected with NLRP3-PYD (**F**) or NLRP3ΔLRR (**G**). Next, 160 µM nitroxoline or an equivalent volume of solvent control was added as indicated. Pronase (28 ng µg^−1^) was added for 30 min, and digestion levels were analyzed by immunoblotting. GAPDH was used as a loading control. Global (**H**) and pose view (**I**) of molecular docking analysis between NLRP3 (PDB: 6NPY) presented as a cartoon model and the nitroxoline molecule as a stick model. Red dashed lines indicate hydrogen bonds, the orange dashed line π-π interactions, and yellow dashed lines van-der-Waals interactions. **J** Quantification of ASC Specks per nucleus of HEK293 cells co-transfected with ASC-Myc and either NLRP3-GFP (WT), NLRP3-GFP-D272N, NLRP3-GFP-F297A, or NLRP3-GFP-R335A. 120 µM nitroxoline or an equivalent volume of solvent control was added as indicated. Data are presented as mean + SD. Data were analyzed using Student’s *t*-test. *p < 0.05.

Nitroxoline is a specific inhibitor of the NLRP3 inflammasome, as it did not reduce IL-1β secretion after activation of the AIM2 inflammasome using LPS and polydA:dT (Fig. 5A) or NLRC4 inflammasome after treatment with LPS and NeedleTox (Fig. 5B). Additionally, pyroptosis measured by PI fluorescence was also not reduced in response to AIM2 or NLRC4 inflammasome activation (Supplementary Fig. 1B, C). Treatment of LPS- and nigericin-challenged THP-1 ASC-GFP cells with nitroxoline disrupted the NLRP3-ASC protein interaction (Fig. 5C). Therefore, drug affinity-responsive target stability assays (DARTs) were performed to identify the target domain of nitroxoline on NLRP3. In this assay, protein lysates are digested with pronase, a mixture of non-specific proteases. The resulting protein digestion pattern was analyzed by immunoblotting. The addition of a small molecule prior to pronase-mediated digestion results in fewer protein fragments if a direct interaction occurs [23, 27]. HEK293 cells were transfected with plasmids encoding NLRP3, NLRP3-PYD containing only the PYD domain, or NLRP3ΔLRR containing the PYD and NACHT domains (Fig. 5D). Preincubation with 80 µM nitroxoline partially protected full-length NLRP3 from pronase-induced degradation, and this effect was more pronounced at 160 µM nitroxoline (Fig. 5E). To highlight potential differences in the degradation pattern, 160 µM nitroxoline was used in subsequent DARTs experiments. Preincubation with nitroxoline did not protect the PYD domain from pronase-mediated degradation (Fig. 5F). However, fewer NLRP3 fragments were detected when nitroxoline was added to the lysates from cells expressing the NLRP3ΔLRR construct (Fig. 5G), suggesting that nitroxoline binds directly to the NACHT domain and shields portions of it from pronase digestion. Molecular docking analysis revealed that nitroxoline may interact with arginine 335 and glycine 271, both situated in the NACHT domain, by forming hydrogen bonds. π-π interactions were observed between the benzene ring of nitroxoline and phenylalanine 297, as well as van der Waals interactions between nitroxoline and isoleucine 257, leucine 270, and aspartic acid 272 (Fig. 5H, I). Mutation of aspartic acid to asparagine (D272N), phenylalanine to alanine (F297A), and arginine to alanine (R335A) resulted in overall lower inflammasome activity and complete abolishment of the inhibitory effect of nitroxoline (Fig. 5J).

## Discussion

The main finding of this study was the identification of nitroxoline as an efficient inhibitor of the NLRP3 inflammasome. Nitroxoline significantly reduced pyroptosis by disrupting inflammasome assembly through direct interaction with the NLRP3 protein, resulting in a reduction of the secretion of the pro-inflammatory cytokine IL-1β by more than 90% in THP-1 monocytes. These effects were confirmed in freshly isolated human PBMC.

Anti-inflammatory strategies are of increasing interest in cardiovascular disease. The most prominent example is the positive CANTOS trial, which used IL-1β inhibition by Canakinumab [28]. The NLRP3 inflammasome is an attractive target since it is located upstream of IL-1β release in the inflammatory cascade. Recently, application of low-dose colchicine has been included in the European Guidelines for the secondary prevention of cardiovascular events. By stabilizing microtubules, colchicine interferes with the approximation of NLRP3 and ASC via α-tubulin, functioning as an indirect inhibitor of NLRP3 [29]. To date, no specific NLRP3 inhibitors have been approved for use in humans. Nitroxoline functions as an inhibitor of cathepsin B, thereby indirectly suppressing NLRP3 inflammasome activation. However, a direct interaction between nitroxoline and the NLRP3 inflammasome has not yet been demonstrated [30]. Such an interaction is of particular interest, as nitroxoline is already clinically used to treat urinary tract infections and is generally well tolerated [31].

Co-immunoprecipitation of ASC-GFP and NLRP3 revealed an inhibitory effect of nitroxoline before the binding step of these two proteins, hinting at an inhibitory effect during NLRP3 activation. The DARTs assay confirmed a direct interaction between nitroxoline and the NLRP3-NACHT domain. This finding was supported by molecular docking analysis, which suggested that nitroxoline interacts with R335 and G271 via hydrogen bonds. Both amino acids are located in the NBD domain of the NACHT domain. By binding to this region, nitroxoline could disrupt the NBD-mediated “head-face” interaction between the NACHT and LRR domains of two NLRP3 proteins, disrupting NLRP3 octamer formation and subsequent inflammasome assembly [32]. Mutation of amino acids within the binding site, identified through molecular docking, led to a reduction in NLRP3 inflammasome activity. Moreover, nitroxoline showed no inhibitory effect on these mutant constructs, indicating that the binding site is functionally relevant and mediates inhibition when it is altered. Although NLRC4 and NLRP3 share substantial homology within the NBD domain [32], nitroxoline did not inhibit NLRC4-mediated IL-1β release, suggesting specificity for NLRP3.

The NF-κB signaling pathway was not affected by nitroxoline. There were no significant changes in IκBα phosphorylation or p65 nuclear translocation, indicating that nitroxoline does not interfere with the priming step of inflammasome activation. Our data show that nitroxoline does not affect NLRC4, which detects bacterial components. This suggests that the immune response to bacterial infections in treated patients is likely preserved and may even be supported by the nitroxoline’s antibiotic properties. Nitroxoline is one of the few antibiotics approved for long-term use and has demonstrated a very low potential to induce bacterial resistance [31, 33, 34]. These advantages make nitroxoline a promising therapeutic option for NLRP3-related diseases such as gout, CAPS syndrome, and potentially atherosclerosis. Short-term inhibition of the NLRP3 inflammasome could theoretically be beneficial in acute inflammatory events such as acute myocardial infarction. During myocardial infarction, mitochondrial damage and generation of reactive oxygen species activate the NLRP3 inflammasome upon reperfusion [35]. The resulting pyroptosis contributes to secondary cell death and enlargement of the infarct area, potentially leading to worse clinical outcomes [36]. The role of NLRP3 in reperfusion injury is well characterized in mouse models, where its inhibition has been shown to reduce infarct size during reperfusion [37]. Recent findings from our group showed that pyroptosis and ASC speck release are increased in patients with cardiogenic shock and are further exacerbated by reperfusion [38].

In summary, these data suggest that nitroxoline is a novel NLRP3-specific inhibitor of pyroptosis that interferes with inflammasome assembly, thereby effectively reducing IL-1β release. Combined with promising findings on toxicity, tolerability, and safety, these results provide a strong rationale for testing the effects of nitroxoline on NLRP3 inflammasome activation in clinical trials involving patients with chronic inflammatory diseases.

## Materials and methods

### Medium-throughput screening for pyroptosis inhibitors

Inhibition of pyroptosis was detected by a reduction in propidium iodide (PI) fluorescence in LPS- and nigericin-treated THP1 ASC-GFP cells. 5 × 10^3^ cells were dispensed per well containing 20 µM compound and 1 µg ml^−1^ LPS for 3 h before the addition of 5 µg ml^−1^ nigericin and 1 µg ml^−1^ propidium iodide for 1 h. Compounds from the Bioactive Compound Library (Selleckchem, TX, USA) and Spectrum Library (MS Discoveries, CT, USA) were used for medium-throughput screening. PI fluorescence intensity was measured at 620/10 nm using a plate reader (POLARstar Omega; BMG Labtech, Germany). The cutoff was set for fluorescence values that were two standard deviations below the mean value of the plate. Primary hits were filtered according to their commercial availability, and compounds with previously reported toxicity were excluded.

### Cell lines and treatment

Monocytic THP-1 (ACC16, German Collection of Microorganisms and Cell Cultures GmbH (DSZM, Germany)) and THP-1 ASC-GFP (thp-ascgfp, Invivogen, CA, USA) cell lines were used throughout the experiments. THP-1 cells were cultured in RPMI-1640 (Merck, Germany) with 10% fetal calf serum (Merck, Germany), 1% penicillin-streptomycin (100 units/L; Lonza, Switzerland), and 25 mM HEPES solution (Merck, Germany). THP-1 ASC-GFP cells were cultured in THP-1 medium with 100 µg ml^−1^ normocin (Invivogen, CA, USA) and 100 µg ml^−1^ zeocin (Invivogen, CA, USA). HEK-293 cells were cultured in DMEM-F12 (Thermo Fisher Scientific, MA, USA) medium supplemented with 10% fetal calf serum and 1% penicillin-streptomycin.

### Inflammasome activation

In general, THP-1 cells were primed for 3 h with 1 µg ml^−1^ LPS (Merck, Germany) and 80 µM nitroxoline (Selleckchem, TX, USA) or volume equivalent solvent control (DMSO; Carl Roth, Germany) before treatment with 5 µg ml^−1^ nigericin (Merck, Germany) for 1 h to activate the NLRP3-inflammasome. AIM-2 inflammasome activation was induced by cell priming for 4 h with LPS and nitroxoline or solvent control. Subsequently, the cells were transfected with 3 µg polydA:dT (Invivogen, CA, USA) using Lipofectamine 2000 (Thermo Fisher Scientific, USA) and incubated overnight. NLRC4 inflammasome activation was performed by LPS-priming for 4 h with nitroxoline or DMSO. Bacillus anthracis protective antigen (20 ng ml^−1^; Merck, Germany) and LFn-Needle (1 µg ml^−1^; NeedleTox; Invivogen, CA, USA) were added to the cells and incubated overnight.

### Cytotoxicity and cell viability assays

Cytotoxicity was measured using the CellTiter-Glo Assay (Promega, WI, USA). 0.04 × 10^6^ THP-1 or 0.1 × 10^6^ PBMC were treated with nitroxoline or volume-equivalent dimethylsulfoxide (DMSO) at the indicated concentrations for 4 h. The assay was performed according to the manufacturer’s instructions, and luminescence was detected using Varioscan LUX (Thermo Fisher Scientific, MA, USA).

### Caspase-1 inhibition assay

Activated caspase 1 was detected using the FAM-Flica Caspase-1 (YVAD) Assay kit (ImmunoChemistry Technologies, CA, USA). The assay was performed according to the manufacturer’s instructions. In brief, 0.2 × 10^6^ THP-1 cells were treated with 80 µM nitroxoline and primed with LPS for 3 h. Equivalent volumes of DMSO were used as the solvent control. Afterward, 5 µg ml^−1^ nigericin was added with 150x Flica solution in a 1:5 ratio for 1 h. Cells were washed thrice and incubated with 2.5 µl 7AAD for 10 min. Cells were analyzed using BD FACSLyric flow cytometer (BD Biosciences, NJ, USA).

### Plasmids

pcDNA3-Myc-ASC and pEGFP-C2-NLRP3 were a gift from Christian Stehlik (Addgene plasmid # 73952 ; http://n2t.net/addgene:73952 ; RRID:Addgene_73952; Addgene plasmid # 73955 ; http://n2t.net/addgene:73955 ; RRID:Addgene_73955) [39]. The deletion constructs pEGFP-C2-NLRP3-PYD and pEGFP-C2-NLRP3 NLRP3ΔLRR were designed according to Coll et al. [23]. Domains were deleted from the pEGFP-C2-NLRP3 plasmid using In-Fusion Snap Assembly MasterMix (Takara Bio, Japan) following the manufacturer’s instructions (primers are listed in Supplementary Table 1).

### Site-directed mutagenesis

Plasmids encoding the mutant NLRP3-GFP variants D272N, F297A, and R335A were generated using the QuikChange II Site-Directed Mutagenesis Kit (Agilent, USA) according to the manufacturer’s instructions with the pEGFP-C2-NLRP3 plasmid described above. The primers used for variant generation are listed in Supplementary Table 1.

### ASC speck formation in HEK cells

HEK293 cells were co-transfected with either pEGFP-C2-NLRP3 or mutant variants D272N, F297A, and R335A, together with pcDNA3-Myc-ASC using polyethyleneimine reagent (PolyScience, PA, USA) at a 1:3 DNA:PEI ratio. After 4 h of transfection, 120 µM nitroxoline or an equivalent volume of DMSO was added and incubated overnight. ASC Specks and nuclei were captured using a Zeiss Elyra super-resolution microscope (Zeiss, Germany) with EC Plan Neofluor 10x/0.30 M27 (Zeiss, Germany) with a 1.6× magnifying lens and LBF 405/488/561/642 filters. The nuclei were counterstained with Hoechst and captured using a BP 445/50. ImageJ software was used to quantify ASC specks and nuclei.

### Co-Immunoprecipitation

15 × 10^6^ THP-1 ASC-GFP cells were treated with 120 µM nitroxoline or a volume equivalent of DMSO and primed with 1 µg ml^−1^ LPS, followed by the addition of 5 µg ml^−1^ nigericin 1 h before cell harvest. Cells were lysed using hypotonic lysis buffer (20 mM Hepes, 10 mM KCl and 1 mM EDTA, pH 7.4) supplemented with a Halt Protease-Inhibitor-Cocktail (Thermo Fisher Scientific, MA, USA). Co-immunoprecipitation was performed using the ChromoTek GFP-Trap Agarose Kit (Proteintech, IL, USA) according to the manufacturer’s instructions. Anti-NLRP3 (D4D8T; Cell Signaling Technology, MA, USA) and anti-ASC B3 (sc-514414; Santa Cruz Biotechnology, Inc., TX, USA) antibodies were used for immunoblotting.

### Drug affinity responsive target stability (DARTs) assay

DARTs assay was performed according to published protocols [27]. In brief, HEK293 cells were transfected with pEGFP-C2-NLRP3, pEGFP-C2-NLRP3-PYD, or pEGFP-C2-NLRP3 NLRP3ΔLRR plasmid using PEI as described above. Cells were harvested 24 h post-transfection, and proteins were isolated using DARTs Lysis Buffer (M-PER reagent (Thermo Fisher Scientific, MA, USA) supplemented with 50 mM sodium fluoride (Carl Roth, Germany), 10 mM β-glycerophosphate Carl Roth, Germany), 5 mM sodium pyrophosphate (Sigma Aldrich, USA), and 20 mM sodium orthovanadate (Merck, Germany). The cell suspension was centrifuged for 10 min at 18,000 × *g*, and the supernatant was transferred to a fresh tube. TNC buffer (500 mM Tris-HCl (Carl Roth, Germany), 500 mM sodium chloride (Carl Roth, Germany), and 100 mM calcium chloride (Carl Roth, Germany) diluted in ultrapure water) was added. Protein lysates were incubated with the indicated compounds or the corresponding solvent control for 1 h at room temperature. 14 ng Pronase (Roche, Switzerland) was added per 50 µg of protein and incubated for 30 min at room temperature. The digestion was abrogated by adding a 20x protease inhibitor cocktail (Merck, Germany). Samples were analyzed by immunoblotting to detect NLRP3 (D4D8T, Cell Signaling Technology, MA, USA; Cryo-2, Adipogen, CA, USA) and GAPDH as loading control (clone 0411, Santa Cruz Biotechnology, Inc., TX, USA).

### Immunostaining

P65 nuclear translocation was analyzed in THP-1 macrophages. Differentiation was induced by adding 100 nM phorbol-12-myristate-13-acetate (PMA; tlrl-pma, InvivoGen, CA, USA) to the culture medium for three days. Afterward, the culture medium was changed to a starving medium containing 1% BSA (Serva, Germany) instead of FCS. THP-1 macrophages were treated with 80 µM nitroxoline and primed with LPS for 3 h. Equivalent volumes of DMSO were used as solvent control. Cells were then fixed using Roti Histofix (Carl Roth, Germany) for 15 min. For immunostaining, cells were permeabilized using 0.1% Triton-X-100 (Merck, Germany) in PBS for 10 min and blocked in 10% donkey serum (Merck, Germany) for 1 h. A primary NF-κB p65 antibody (D14E12, Cell Signaling Technology, MA, USA) was diluted 1:400 and incubated overnight. Donkey anti-Rabbit 488 (A-21206, Invitrogen, CA, USA) was added the next day, and nuclei were counterstained using Hoechst. High-resolution images were captured using a Zeiss Elyra super-resolution microscope (Zeiss, Germany) with Plan Apochromat 20x/0.8 M27 (Zeiss, Germany) with a 1.6× magnifying lens and BP 495-550 + BP 570-620 filters. Hoechst was captured using a BP 445/50. Images for quantitative analysis were captured using a Keyence BZ-X810 fluorescence microscope with a 10x PlanApoλ NA 0.45 lens (Keyence, Japan). ImageJ software was used for p65 nuclear translocation analysis, following the protocol described by Wessel and Hanson [40].

### Protein preparation and immunoblot

Protein lysates were prepared using 1X RIPA buffer (Merck, Germany) supplemented with HALT protease and phosphatase inhibitor (Thermo Fisher Scientific, MA, USA). Immunoblot was performed as previously described [41]. Ikba (#4814S, Cell Signaling Technology, MA, USA) and pIkba (#9246S, Cell Signaling Technology, MA, USA) antibodies were applied to determine the pIkba/Ikba ratio. Gasdermin D antibody (#39754S, Cell Signaling Technology, MA, USA) was used to detect gasdermin full-length and cleaved gasdermin-NT. A β-actin antibody (#WA-AM1829B, Biomol, Germany) was applied for the detection of the loading control protein.

### Isolation of peripheral blood mononuclear cells

Human peripheral blood mononuclear cells (PBMC) were isolated from buffy coats of healthy volunteers via Ficoll (Cytiva, MA, USA) gradient centrifugation. Isolated PBMC were cultured in RPMI-1640 supplemented with 10% FCS and 1% penicillin-streptomycin.

### Flow cytometric analysis of apoptosis-associated speck-like proteins specks in THP-1 ASC-GFP cells

THP-1 ASC-GFP cells were treated with 80 µM nitroxoline or DMSO and primed with 1 µg ml^−1^ LPS for 3 h. 5 µg ml^−1^ nigericin and propidium iodide were then added for 1 h. Cells were harvested and washed twice with pre-warmed FACS buffer (PBS supplemented with 1% BSA and 0.05% sodium azide (Merck, Germany) before analysis at the BD FACSLyric flow cytometer. Gating was performed as described by Sester et al. [42].

### Molecular docking analysis

Molecular docking analysis was performed by Creative Proteomics (Shirley, NY, USA).The NLRP3 protein was extracted from the PDB database (PDB: 6NPY). The PDB structure was downloaded and used as the initial structure of the protein receptor. The initial structure was processed with AutoDock Tools 1.5.6 [43], which included hydrogen addition, charge preservation, docking atom types assignation, and pdbqt file generation for docking. The ligand nitroxoline was searched by CID number (CID: 19910) to obtain mol structure, which was then converted into three-dimensional molecular structures using OpenBabel 2.3.1 [44]. The MMFF94 force field was used to optimize the three-dimensional structure of the ligand. Finally, AutoDock Tools 1.5.6 was used to process the ligand structures, including hydrogen addition, charge calculation, docking atom types assignation, and pdbqt files generation for docking. Docking was implemented with AutoDock vina1.2.3 [45, 46]. The docking box center coordinates were set in the main structural region of the protein with a grid size of 0.75 Å, covering all possible binding sites to find the optimal binding region. Subsequently, a grid size of 0.375 Å was used for docking all small molecules in the optimal binding region. Default values were used for other parameters. The interactions between the small molecule and the target protein were analyzed using pymol 2.5.0 software, and visualized using Pymol [47].

### ELISA

Human IL-1β ELISA kit (R&D Systems, MN, USA) was used in combination with DuoSet ELISA Ancillary Reagent kit (R&D Systems, MN, USA). The assay was performed following the manufacturer’s instructions.

### Statistics

Statistical analyses were performed using GraphPad Prism (version 8; GraphPad Software Inc., La Jolla, CA, USA). Data were tested for a Gaussian distribution using the Kolmogorov–Smirnov or D’Agostino-Pearson normality tests. A two-tailed unpaired t-test was performed to compare the groups, unless otherwise stated. The significance level was set to *p* < 0.05.

## Supplementary information

Supplementary figure legends

Supplementary table 1

Supplemental Figure 1

Supplemental Figure 2

Western Blots uncropped

## Data Availability

Datasets generated and/or analyzed during the current study are available from the corresponding author upon reasonable request.
