# Supplementary material for: Nitroxoline is a novel inhibitor of NLRP3-dependent pyroptosis
Source: Cell Death Discov. 2025 Aug 20;11:394. doi: 10.1038/s41420-025-02699-z (PMC12368067; doi:10.1038/s41420-025-02699-z)
Supplement: Supplementary file 1 — Supplementary figure legends [file 41420_2025_2699_MOESM1_ESM.docx]

**Supplementary data**

**Supplementary Figure 1:**

A: Bar plot presenting the fold change in IkBa protein levels compared to the control (n=4). **B, C:** Percentage of Propidium iodide (PI)-positive THP-1 WT cells treated with LPS and polydA:dT (n=3, **panel B**) or LPS and NeedleTox (n=3, **panel C**).

**Supplementary Figure 2:**

**A:** Representative images of ASC Specks per nucleus of HEK293 cells co-transfected with ASC-Myc and either NLRP3-GFP (WT), NLRP3-GFP-D272N, NLRP3-GFP-F297A, or NLRP3-GFP-R335A. 120 µM nitroxoline or an equivalent volume of solvent control was added as indicated. Cells were imaged at 16 × magnification, with a scale bar of 100 µm. NLRP3-GFP is shown in green and nuclei are shown in blue.

**Supplementary Table 1**

Primers used for site-directed mutagenesis and deletion constructs
