# Supplementary material for: Nitroxoline is a novel inhibitor of NLRP3-dependent pyroptosis
Source: Cell Death Discov. 2025 Aug 20;11:394. doi: 10.1038/s41420-025-02699-z (PMC12368067; doi:10.1038/s41420-025-02699-z)
Supplement: Supplementary file 2 — Supplementary table 1 [file 41420_2025_2699_MOESM2_ESM.docx]

| **Primer** | **Sequence (5‘ to 3‘)** |
| --- | --- |
| R335A_fw | CTCGGGAAGCAGCTTCTTTGCGATGAGGCTGCTCAGGAG |
| R335A_rev | CTCCTGAGCAGCCTCATCGCAAAGAAGCTGCTTCCCGAG |
| F297A_fw | CGAAGCCGTCCATGAGGGCGAGGATTCTGGAGGGTT |
| F297A_rev | AACCCTCCAGAATCCTCGCCCTCATGGACGGCTTCG |
| D272N_fw | GCAGCTCATGATCAGGTTCCCCAGGCTCCTCTG |
| D272N_rev | CAGAGGAGCCTGGGGAACCTGATCATGAGCTGC |
| delLRR_fw | CCAGCCAGGTGCTGAAACAGCAGAGCTGC |
| delLRR_rev | TCAGCACCTGGCTGGTGCTCAGAACTG |
| NLRP3-PYD_fw | AGCCGAAGGTGCTGAAACAGCAGAGCTGC |
| NLRP3-PYD_rev | TCAGCACCTTCGGCTCATCTCTTTTTGCTTTC |
