# Supplementary figures and images for: Nitroxoline is a novel inhibitor of NLRP3-dependent pyroptosis
Source: Cell Death Discov. 2025 Aug 20;11:394. doi: 10.1038/s41420-025-02699-z (PMC12368067; doi:10.1038/s41420-025-02699-z)

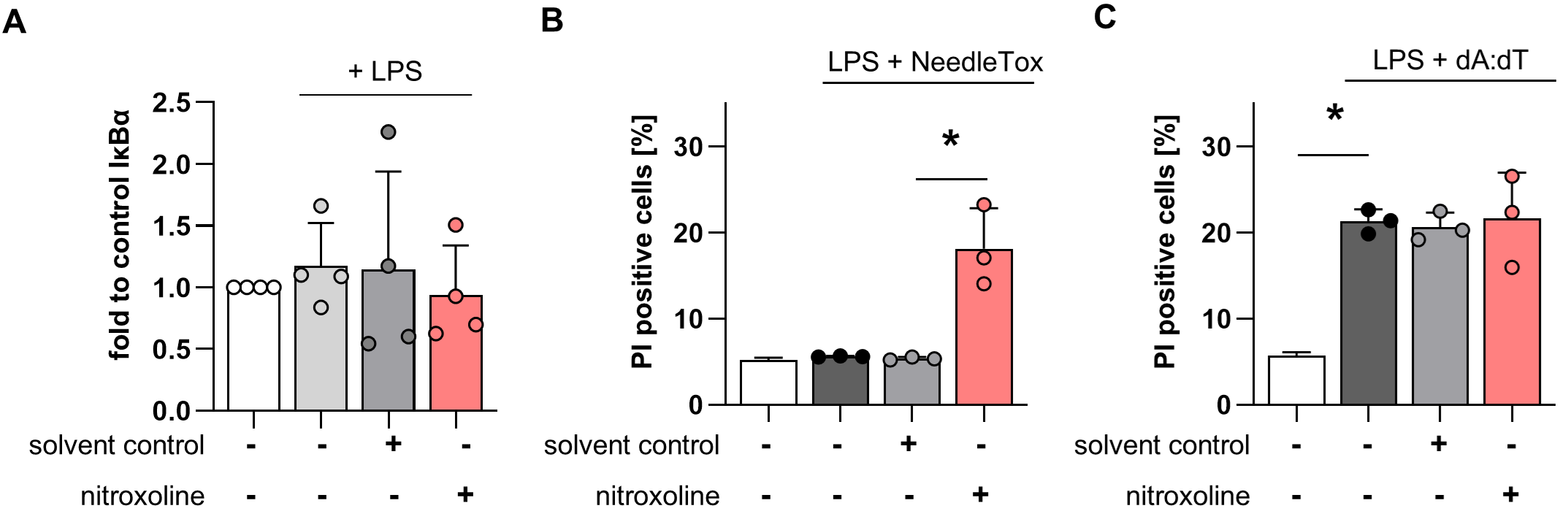

Supplement: Supplementary file 3 — Supplemental Figure 1 [file 41420_2025_2699_MOESM3_ESM.png]

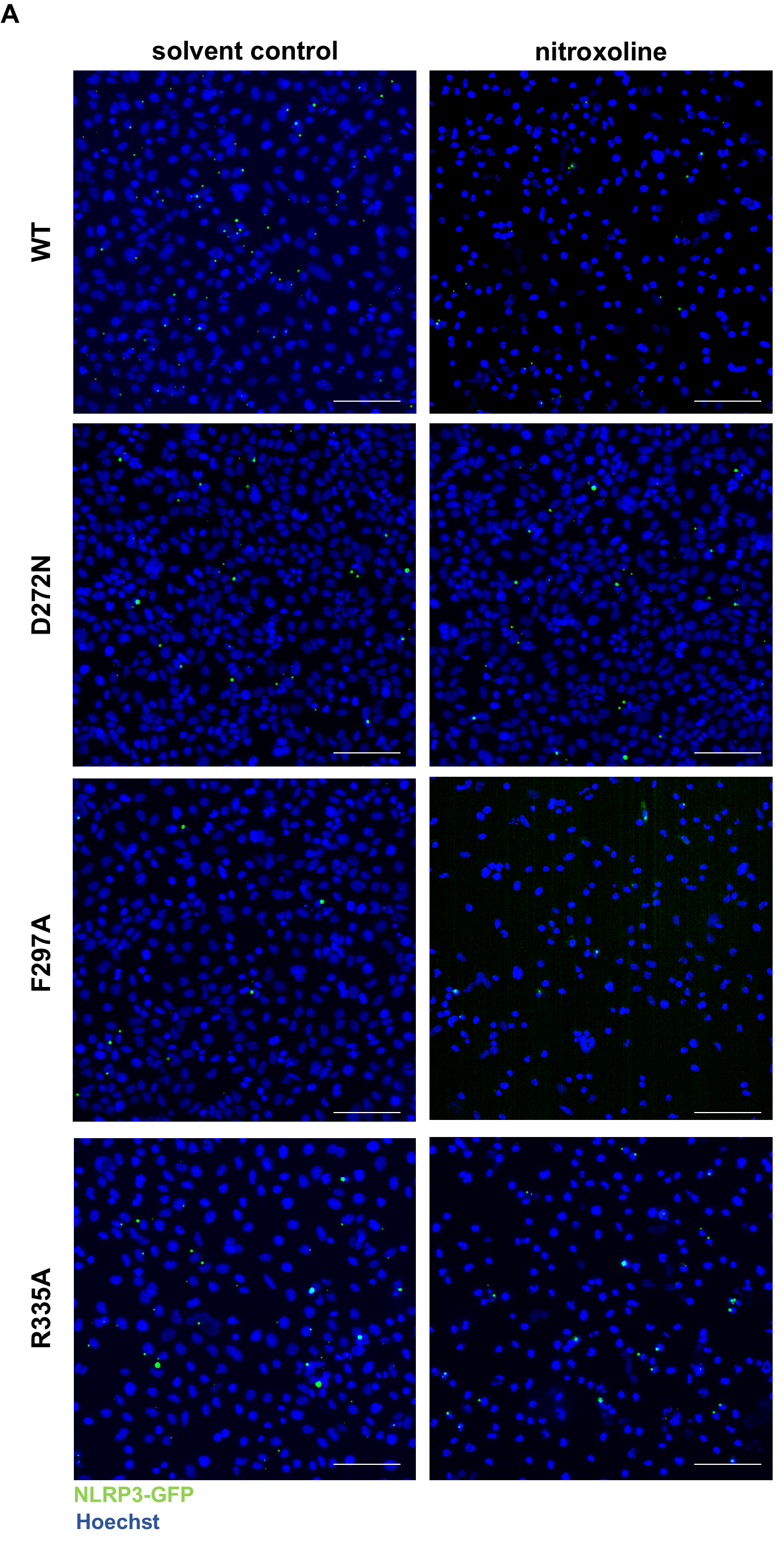

Supplement: Supplementary file 4 — Supplemental Figure 2 [file 41420_2025_2699_MOESM4_ESM.png]
