# Supplementary material for: Nitroxoline is a novel inhibitor of NLRP3-dependent pyroptosis
Source: Cell Death Discov. 2025 Aug 20;11:394. doi: 10.1038/s41420-025-02699-z (PMC12368067; doi:10.1038/s41420-025-02699-z)
Supplement: Supplementary file 5 — Western Blots uncropped [file 41420_2025_2699_MOESM5_ESM.pptx]

## Slide 1
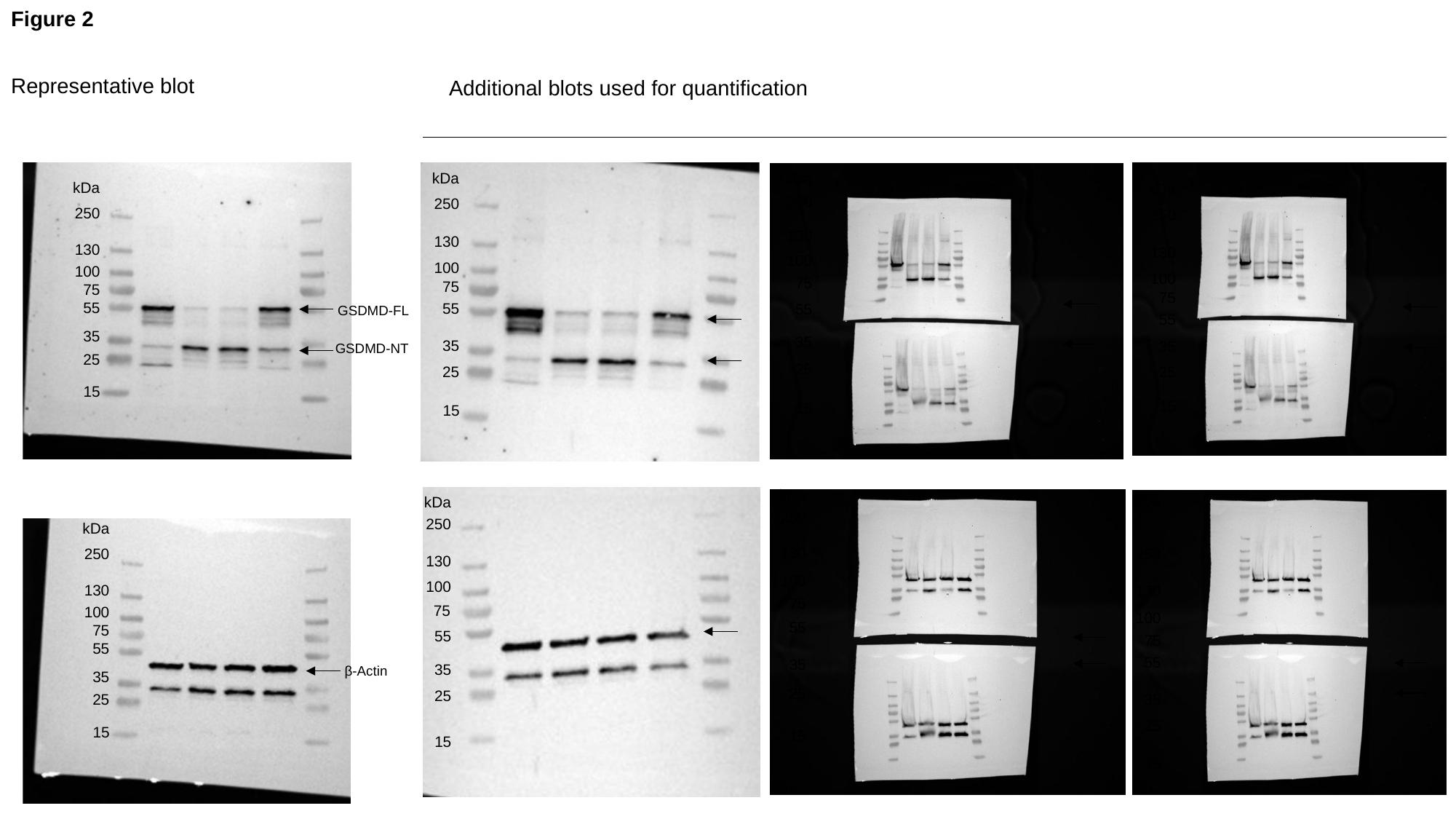

Figure 2
Representative blot
Additional blots used for quantification
kDa
kDa
kDa
kDa
250
250
250
250
130
130
130
130
100
100
100
100
75
75
75
75
55
55
55
GSDMD-FL
55
35
35
35
35
GSDMD-NT
25
25
25
25
15
15
15
15
kDa
kDa
kDa
250
250
kDa
130
250
250
130
100
100
130
130
75
75
100
100
55
75
55
75
55
55
35
35
β-Actin
35
25
25
35
25
25
15
15
15
15

## Slide 2
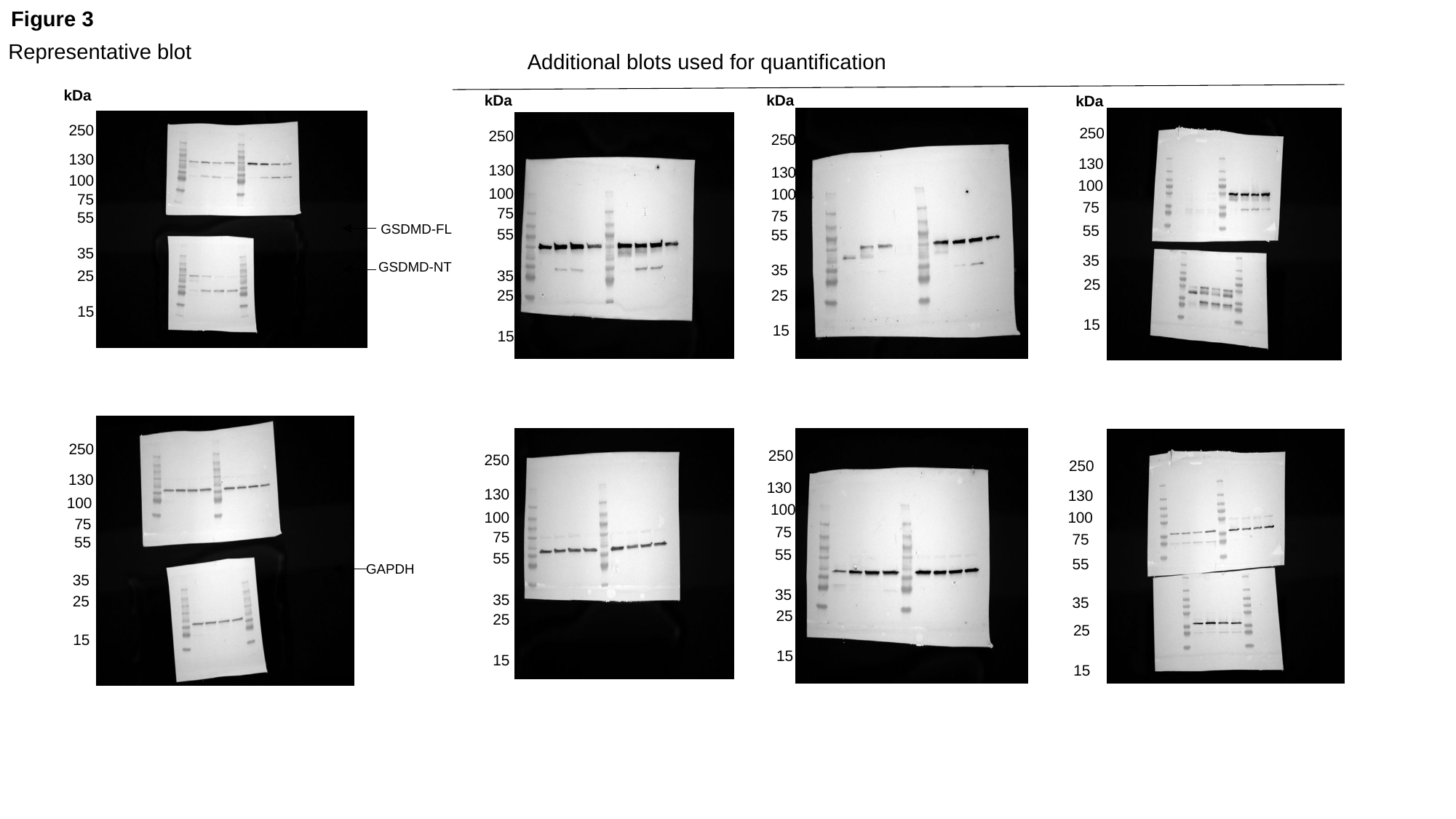

Figure 3
Representative blot
Additional blots used for quantification
kDa
kDa
kDa
kDa
250
250
250
130
250
130
130
130
130
100
100
100
100
75
75
75
75
55
GSDMD-FL
55
55
55
35
35
GSDMD-NT
35
25
35
25
25
25
15
15
15
15
250
250
250
250
130
130
130
130
100
100
100
100
75
75
75
75
55
55
55
55
GAPDH
35
35
35
25
35
25
25
25
15
15
15
15

## Slide 3
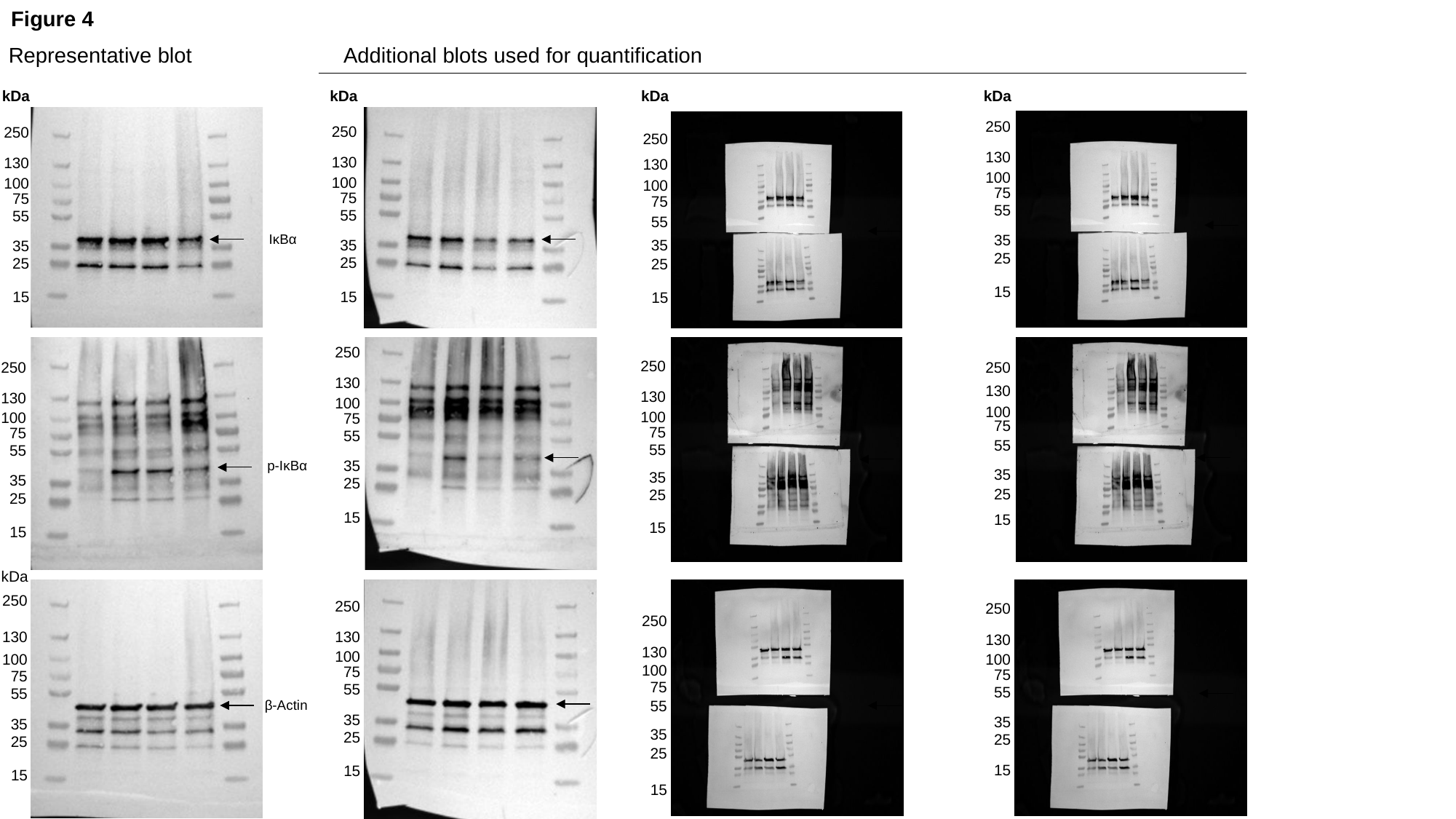

Figure 4
Representative blot
Additional blots used for quantification
kDa
kDa
kDa
kDa
250
250
250
250
130
130
130
130
100
100
100
100
75
75
75
75
55
55
55
55
IκBα
35
35
35
35
25
25
25
25
15
15
15
15
250
250
250
250
130
130
130
130
100
100
100
100
75
75
75
75
55
55
55
55
35
p-IκBα
35
35
35
25
25
25
25
15
15
15
15
kDa
250
250
250
250
130
130
130
130
100
100
100
100
75
75
75
75
55
55
55
55
β-Actin
35
35
35
35
25
25
25
25
15
15
15
15

## Slide 4
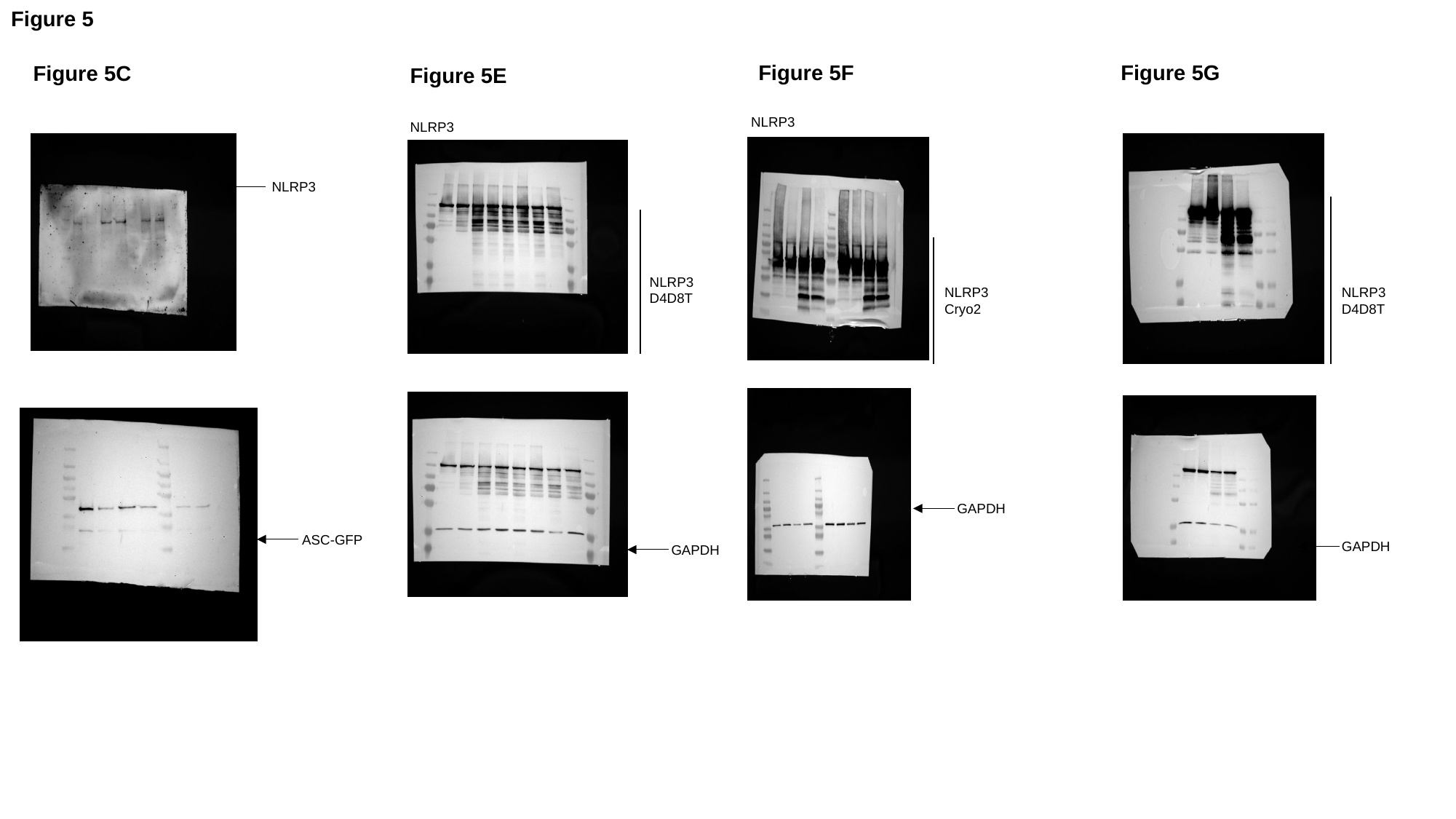

Figure 5
Figure 5G
Figure 5F
Figure 5C
Figure 5E
NLRP3
NLRP3
NLRP3
NLRP3
D4D8T
NLRP3
Cryo2
NLRP3
D4D8T
GAPDH
ASC-GFP
GAPDH
GAPDH
